# A case report of complement *C4B* deficiency in a patient with steroid and IVIG-refractory anti-NMDA receptor encephalitis

**DOI:** 10.1186/s12883-020-01906-x

**Published:** 2020-09-08

**Authors:** Gilbert T. Chua, Danlei Zhou, Alvin Chi Chung Ho, Sophelia Hoi Shan Chan, Chack Yung Yu, Yu Lung Lau

**Affiliations:** 1 Department of Paediatrics and Adolescent Medicine, Queen Mary Hospital, The University of Hong Kong, Room 106, 1/F, New Clinical Building, 102 Pokfulam Road, Pokfulam, Hong Kong; 2 Center for Microbial Pathogenesis and Division of Rheumatology, Abigail Wexner Research Institute at Nationwide Children’s Hospital and Department of Pediatrics, The Ohio State University, 700 Children’s Drive, Columbus, OH 43205 USA

**Keywords:** Anti-NMDA receptor encephalitis, Homozygous C4B deficiency, Plasmapheresis

## Abstract

**Background:**

Complement C4A or C4B deficiency has never been reported in autoantibody-associated encephalitides patient. Here we present a case of anti-*N*-methyl- D-aspartate (NMDA) receptor encephalitis associated with homozygous C4B deficiency, who did not respond to intravenous immunoglobulin and pulse methylprednisolone but plasmapheresis and rituximab.

**Case presentation:**

A fourteen-year-old boy presented to our unit with subacute onset of behavioral changes and confusion, and was later confirmed to be anti-NMDA receptor encephalitis. He was initially managed with intravenous immunoglobulin (IVIG) and pulse methylprednisolone but did not achieve any clinical improvement. Seven sessions of plasmapheresis was commenced with remarkable improvement after the second session, and was followed by four doses of rituximab. His neurological and cognitive functioning gradually returned to baseline. Immunological investigations demonstrated persistently low C4 levels below 8 mg/dL. A more in-depth complement analysis of the patient and his family showed that he has homozygous C4B deficiency. Genetic analysis revealed that the index patient has homozygous deficiency in complement C4B and he carries one non-functioning mutant *C4B* gene inherited from his mother.

**Conclusions:**

Low levels of serum C4 correlate with reduced functions of the classical and lectin pathways, leading to the impairment of immune-complexes removal. Plasmapheresis ameliorates complement deficiency and removes the offending immune-complexes leading to clinical improvement that was not achieved by IVIG and steroids. We postulate that serum C4 levels may serve as a biomarker for the need of plasmapheresis upfront rather than only after non-response to steroid and IVIG in treating anti-NMDA-receptor encephalitis.

## Background

Complement C4 plays a crucial role in the activation of the classical and lectin complement pathways. It has two isotypes, C4A and C4B. While deficiency of C4A and C4B are detectable at low frequency in healthy populations, they have also been reported to be associated with various autoimmune and inflammatory diseases [1, 2]. However, a C4A or C4B deficiency has never been reported in autoantibody-associated encephalitides patient. Here we report a case of anti-*N*-methyl- D-aspartate (NMDA) receptor encephalitis associated with homozygous C4B deficiency.

## Case presentation

Our patient is a fourteen-year-old boy with background of mild attention-deficit and hyperactive disorder. He has insignificant family history of neurological, psychological or immunological disorders. Preceded by two weeks of viral illness-like prodromal symptoms, he progressively developed hypersomnolence, confused speech with echolalia, self-muttering, dysarthria, mood fluctuation, bilateral upper limbs tremor, and headache. Physical examination was unremarkable, apart from having confusion, inappropriate speech with foul languages, urine and fecal incontinence. Investigations showed normal complete blood count, liver and renal function tests. Autoimmune markers including anti-nuclear antibodies and anti-double stranded DNA antibody were negative. His serum C3 levels were within normal range between 109 and 115 mg/dL, but his C4 levels were persistently below 8 mg/dL (Table 1). MRI brain was normal. Electroencephalogram showed a mild slowing of background activities. Cerebrospinal fluid (CSF) analysis demonstrated a total white cell count of 33 cells/mm^2^, with normal protein and glucose levels. Oligoclonal protein was detected in the CSF. Microbiological investigations including bacterial cultures and viral polymerase chain reactions for herpes simplex virus, varicella zoster virus and enteroviruses were all negative. Anti-NMDA receptor antibodies based on commercial assay (Euroimmune®, Lueback, Germany) were detectable (titer < 1:10) in the CSF but not the serum.

**Table 1:** Complement genetic profiles of the patient and his family members

	Plasma C4 (mg/dL)	Plasma C3 (mg/dL)	RCCX-C4 haplotypes	Total C4 GCN	C4 Long GCN	C4 Short GCN	C4A GCN	C4B GCN*	C4A Protein	C4B Protein	C4 protein Haplotype1	C4 protein Haplotype2
Patient	7.7	118.5	LL / L	3	3	0	2	(1)	A3A3	Q_0_	A3Q_0_	A3
Mother	20.2	106.4	LL / LLS	5	4	1	3	2 (1)	A3A3A3	B2, Q_0_	A3Q_0_	A3A3B2
Father	19.2	167.6	L / L	2	2	0	2	0	A3A3		A3	A3
Maternal Step Brother	10.0	91.1	LL / L	3	3	0	1	2 (1)	A3	B1, Q_0_	A3Q_0_	B1

*GCN* gene copy number, *L* C4 long gene, *S* C4 short gene, *LL* long-long, *LS* long-short, *LLS* long-long-shortm, *A3 C4A* allotype 3

*B1 C4B* allotype 1, *B2* C4B allotype 2, *Q*_*0*_ zero quantity of C4 protein from the corresponding gene;

* C4B GCN in brackets indicates the number of mutant gene

The diagnosis of anti-NMDA receptor encephalitis was made based on the diagnostic criteria published by Graus et al. [3] He was immediately given intravenous immunoglobulin (IVIG) 1 g/kg for on day 2 of admission for two consecutive days followed by pulse methylprednisolone 1 g on day 6 of admission for five days with gradual taper. However, he did not demonstrate any clinical improvement after IVIG and pulse methylprednisolone.

Plasmapheresis was therefore performed for a total of 7 sessions between day 14 and 29 of admission, with sedation required for the initial two sessions. He demonstrated significant improvement after the second session and was able to complete the remaining plasmapheresis without sedations. Nevertheless, his cognitive improvement remained slow and four doses of rituximab at 375 mg/m^2^/dose at weekly intervals were given between day 35 and 56 of admission. Accelerated improvement was observed after the second dose in terms his self-care abilities, behavior and cognitive functions. He was discharged after eight weeks of admission with some residual cognitive impairment requiring rehabilitation.

**Fig. 1:**
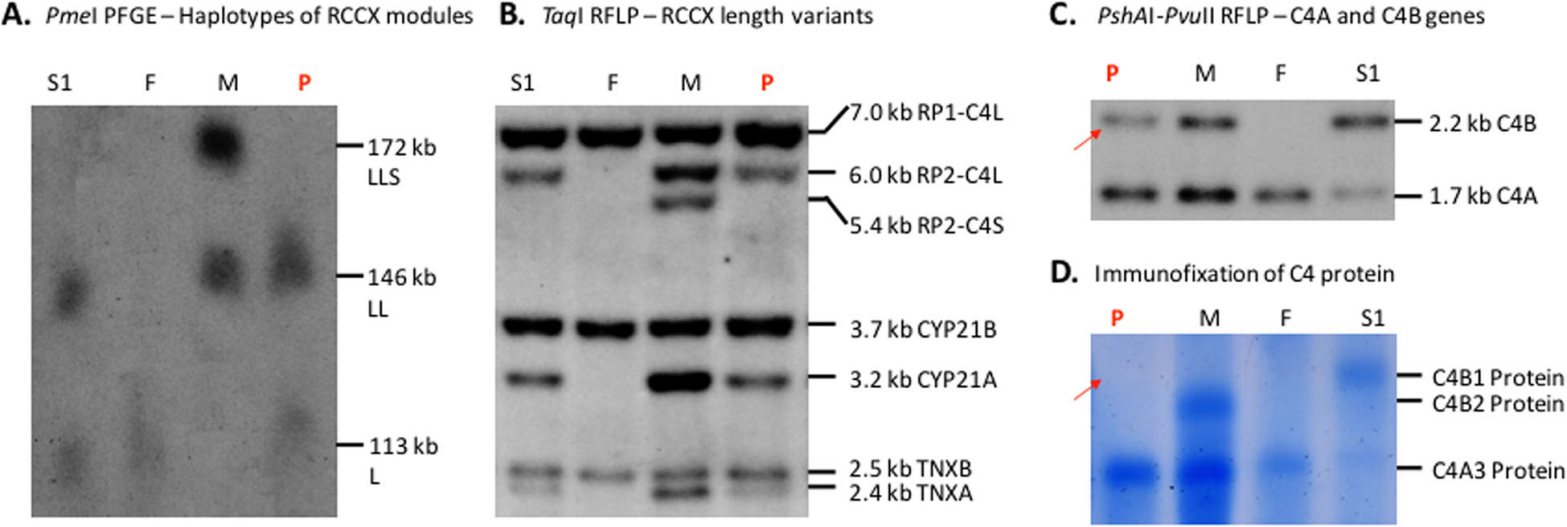
Molecular genetic characterization of the encephalitis patient with antibodies against NMDA-receptor and his family members. **a**. Pulsed field gel electrophoresis (PFGE) of PmeI digested genomic DNA to show RP-C4-CYP21-TNX (RCCX) haplotypes; **b**. TaqI restriction fragment polymorphism (RFLP) to show details of RCCX structures with long (L) and short (S) C4 genes; **c**. PshAI/PvuII RFLP to show the presence and ratios of C4B and C4A genes; **d**. Immunofixation of EDTA-plasma to show polymorphisms of C4A and C4B proteins. A red arrow showed the presence of a C4B gene in panel C but no C4B protein in panel D. Data interpretation is tabulated in Table 1. Abbreviations: P, patient; F, father; M, mother; S1, stepbrother.

In view of his low C4 protein levels, an in-depth genetic analysis of the index patient, his parents and his maternal stepbrother was performed (Fig. 1; Table 1, Supplementary Fig. 1). Long-range mapping by pulsed field gel electrophoresis of *Pme*I-digested genomic DNA revealed heterozygosity with bimodular (LL) and monomodular (L) *RP-C4-CYP21-TNX* (RCCX) haplotypes (*panel* A). Regular Southern blots of *Taq*I digested genomic DNA revealed the presence of three long *C4* genes in LL/L configurations (*panel* B), by which two *C4* genes belong to the *C4A* isotype and one *C4* gene belongs to the *C4B* isotype, as shown by *Psh*AI/*Pvu*II Southern blot (*panel* C). Immunofixation experiment of EDTA-plasma showed that the patient expressed C4A protein but no C4B protein (*panel* D) [4]. In other words, the patient contained a *C4B* gene that did not produce a C4B protein (*panels* C and D, *arrows*) and thus assigned a *C4B* mutant. Concurrent study of the patient’s parents and maternal stepbrother suggested the patient (Fig. 1*,* Table 1) inherited the mutant *C4B* gene originated from their mother with a LL haplotype consisting of one long *C4A* gene coding for C4A3 protein, and one long mutant *C4B* gene with no product.

## Discussion and conclusions

Deficiency of C4B proteins, low gene copy number or mutation of *C4B* gene has not been reported in any autoimmune encephalitides. Therefore, its potential role in the development of anti-NMDA receptor encephalitis remains to be established. Classical pathway complement proteins such as C1q and C4 have been implicated in the wiring of neurons, or the formation and pruning of synapses in the brain [5]. High copy number of *C4A*, which can also be presented as low copy number of *C4B* because a *C4* gene either codes for C4A or C4B protein [2], are associated with schizophrenia [6].

Low levels of serum C4 correlate with reduced functions of the classical and lectin pathways, leading to the impairment of immune-complexes removal [7]. Our patient was a poor responder to IVIG and pulse glucocorticoids but achieved significant improvement after plasmapheresis and rituximab. While a study by Martinez-Hernandez et al. demonstrated that complement-mediated cytotoxicity, i.e. cellular destruction through deposition of C3b and formation of membrane attack complexes C5b-9, was not readily detectable in the pathogenesis of anti-NMDA receptor encephalitis, their study did not examine the activation of early classical pathway complement components such as C1q and C4 [8]. This would be relevant because complement activation on autologous cells are tightly controlled and mostly stop after the deposition of C4b. We postulate that C4B deficiency in our patient might have led to the defective removal of immune complexes and possibly impairment of yet to be characterized neurogenic pathways, which has a substantial role in autoimmune encephalitis [9–11]. Complement deficiency is ameliorated by plasmapheresis, which normally correct the complement deficiency, but not by IVIG. Plasmapheresis also effectively removes offending autoantibodies and immune-complexes [12]. The use of rituximab, an anti-CD20 monoclonal antibodies, depletes CD20^+^ B cells, preventing further autoantibody production and immune complex formation [8]. These strategies lead to a sustained clinical improvement. It remains to be determined if routine plasmapheresis is required to keep patient’s disease in remission.

Further studies investigating the complement profiles and circulating immune complexes in patients with anti-NMDA receptor and other autoimmune encephalitides would clarify the role of *C4B* deficiency. A study by Shu et al. demonstrated that southern Chinese patients with anti-NMDA receptor encephalitis had higher C4 levels among female patients (*N* = 19) than female healthy controls (*N* = 16), the sample sizes (for controls) were relatively small and required validation. Moreover, genetic deficiency of C4B was not examined [13]. While the best first-line therapeutic option (steroid, IVIG or plasmapheresis) to treat anti-NMDA receptor encephalitis remains debatable [14], inheritently low C4 levels or C4B deficiency may be a biomarker for the need of plasmapheresis upfront rather than only after non-response to steroid and IVIG.

## Supplementary information

**Additional file 1.**

## Data Availability

The laboratory data for this study were shown in Table 1 and Fig. 1. Further clinical data would be available from the corresponding author on reasonable request.

## Abbreviations

A3: *C4A* allotype 3
B1: *C4B* allotype 1
B2: C4B allotype 2
CSF: Cerebrospinal fluid
GCN: Gene copy number
IVIG: Intravenous immunoglobulin
NMDA: Anti-*N*-methyl-D-aspartate
L: C4 long gene
LL: Long-long
LS: Long-short
LLS: Long-long-short
S - C4: Short gene.
PFGE: Pulsed field gel electrophoresis
Q_0_: Zero quantity of C4 protein from the corresponding gene
RFLP: Restriction fragment polymorphism

## References

[1] Chen JY, Wu YL, Mok MY (2016). Effects of complement C4 gene copy number variations, size dichotomy, and C4A deficiency on genetic risk and clinical presentation of systemic lupus Erythematosus in east Asian populations. Arthritis Rheumatol.

[2] Lintner KE, Wu YL, Yang Y (2016). Early components of the complement classical activation pathway in human systemic autoimmune diseases. Front Immunol.

[3] Graus F, Titulaer MJ, Balu R (2016). A clinical approach to diagnosis of autoimmune encephalitis. Lancet Neurol.

[4] Chung EK, Wu YL, Yang Y, Zhou B, Yu CY. Human complement components C4A and C4B genetic diversities: complex genotypes and phenotypes. Curr Protoc Immunol*.* 2005;Chapter 13:Unit 13.18. 10.1002/0471142735.im1308s68 18432942

[5] Johnson MB, Stevens B (2018). Pruning hypothesis comes of age. Nature..

[6] Sekar A, Bialas AR, de Rivera H (2016). Schizophrenia risk from complex variation of complement component 4. Nature..

[7] Yang Y, Chung EK, Zhou B (2003). Diversity in intrinsic strengths of the human complement system: serum C4 protein concentrations correlate with C4 gene size and polygenic variations, hemolytic activities, and body mass index. J Immunol.

[8] Martinez-Hernandez E, Horvath J, Shiloh-Malawsky Y, Sangha N, Martinez-Lage M, Dalmau J (2011). Analysis of complement and plasma cells in the brain of patients with anti-NMDAR encephalitis. Neurology..

[9] Bien CG, Vincent A, Barnett MH (2012). Immunopathology of autoantibody-associated encephalitides: clues for pathogenesis. Brain..

[10] Qiu X, Zhang H, Li D, et al. Analysis of Clinical Characteristics and Poor Prognostic Predictors in Patients With an Initial Diagnosis of Autoimmune Encephalitis. Front Immunol. 2019;10(1286). 10.3389/fimmu.2019.01286 PMC6567932 31231392

[11] Magdalon J, Mansur F, Teles E Silva AL, de Goes VA, Reiner O, Sertié AL. Complement system in brain architecture and neurodevelopmental disorders. Front Neurosci 2020;14:23–23. 10.3389/fnins.2020.00023 PMC7015047 32116493

[12] Schwartz J, Padmanabhan A, Aqui N (2016). Guidelines on the use of therapeutic apheresis in clinical practice-evidence-based approach from the writing Committee of the American Society for apheresis: the seventh special issue. J Clin Apher.

[13] Shu Y, Chen C, Chen Y (2018). Serum complement levels in anti-N-methyl-d-aspartate receptor encephalitis. Eur J Neurol.

[14] Shin Y-W, Lee S-T, Park K-I, et al. Treatment strategies for autoimmune encephalitis. Ther Adv Neurol Disord 2017;11:1756285617722347–1756285617722347. 10.1177/1756285617722347 PMC5784571 29399043

