# Supplementary material for: A case report of complement C4B deficiency in a patient with steroid and IVIG-refractory anti-NMDA receptor encephalitis
Source: BMC Neurol. 2020 Sep 8;20:339. doi: 10.1186/s12883-020-01906-x (PMC7488026; doi:10.1186/s12883-020-01906-x)
Supplement: Supplementary file 1 — Additional file 1. [file 12883_2020_1906_MOESM1_ESM.pdf]

Figure 1

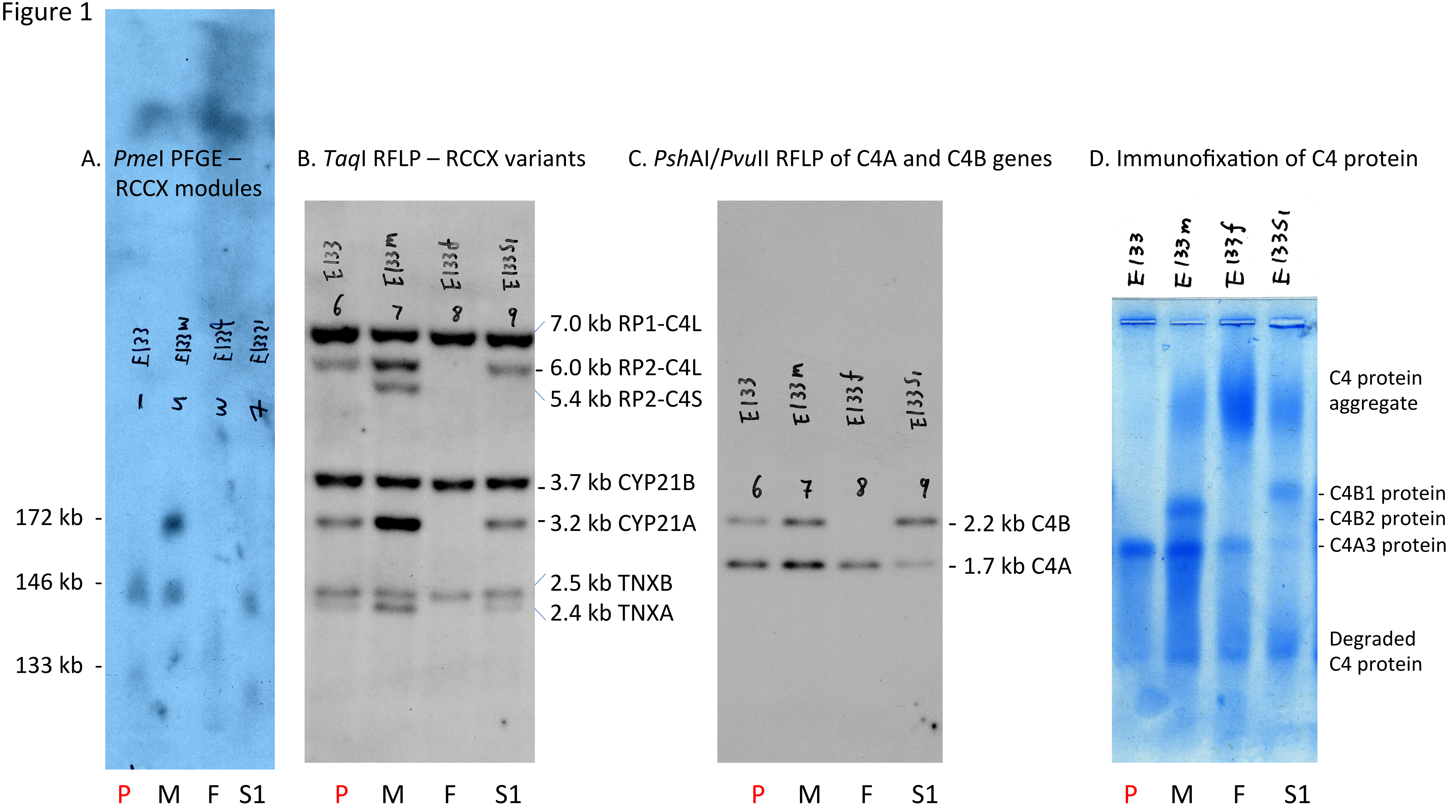
A. PmeI PFGE – RCCX modules
B. TaqI RFLP – RCCX variants
C. PshAI/PvuII RFLP of C4A and C4B genes
D. Immunofixation of C4 protein
172 kb
146 kb
133 kb
7.0 kb RP1-C4L
6.0 kb RP2-C4L
5.4 kb RP2-C4S
3.7 kb CYP21B
3.2 kb CYP21A
2.5 kb TNXB
2.4 kb TNXA
2.2 kb C4B
1.7 kb C4A
C4 protein aggregate
C4B1 protein
C4B2 protein
C4A3 protein
Degraded C4 protein
P M F S1
P M F S1
P M F S1
P M F S1

Legend for Figure 1. Genotypic and phenotypic analyses of the encephalitis patient and family.*

A. Pulsed field gel electrophoresis (PFGE) of *Pme*I digested genomic DNA to show RP-C4-CYP21-TNX (RCCX) haplotypes;

B. *Taq*I restriction fragment polymorphism (RFLP) to show details of RCCX structures with long (L) and short (S) C4 genes;

C. *Psh*AI/*Pvu*II RFLP to show the presence and ratios of C4B and C4A genes;

D. Immunofixation of EDTA-plasma to show polymorphisms of C4A and C4B proteins. A red arrow showed the presence of a C4B gene in panel C but no C4B protein in panel D. Data interpretation is tabulated in Table 1.

Abbreviations: **P**, patient; **F**, father; **M**, mother; **S1**, stepbrother.

Blood samples for this family were taken in Hong Kong and sent to Columbus, OH through express shipment. Partial C4 protein aggregates and partial protein degradation shown in panel D were probably caused by X-ray scans during clearance through US customs and/or the shipment process.

* *Original gels with Southern blots and immunofixed gels are shown in figures of supplementary results.*

Original X-ray films

A. PmeI PFGE

172 kb -

146 kb -

133 kb -

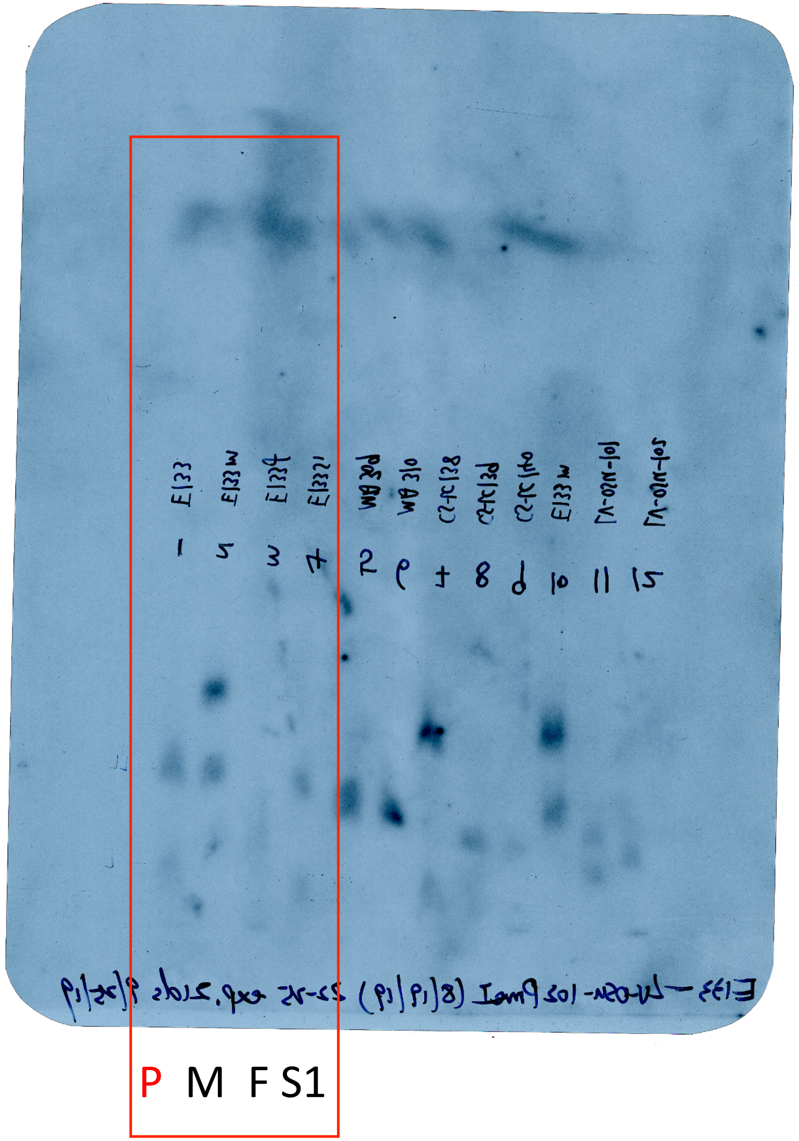

P M F S1

Original X-ray films

B. TaqI restriction length polymorphism (RFLP)

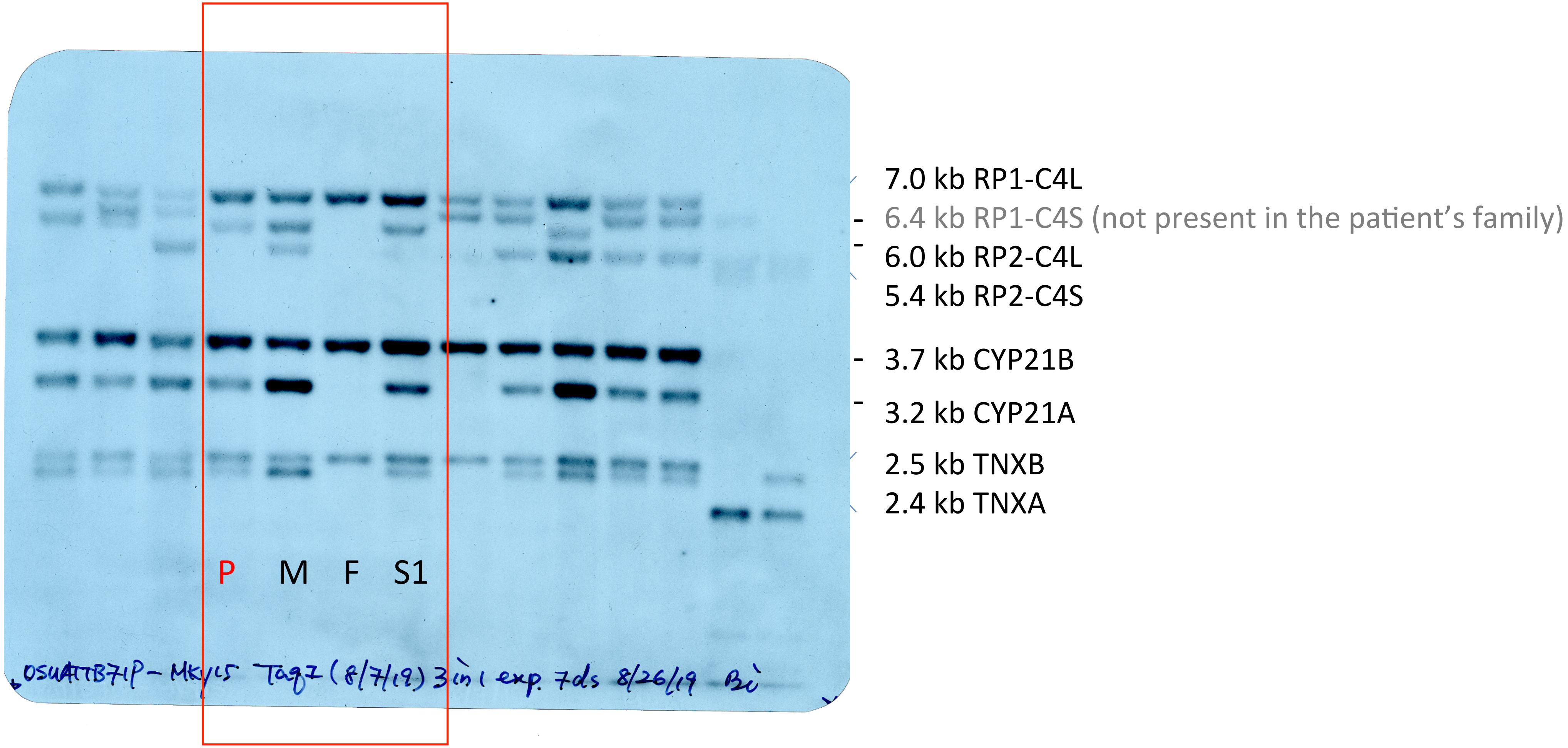

Original X-ray films

C. PshAI/PvuII restriction length polymorphism (RFLP)

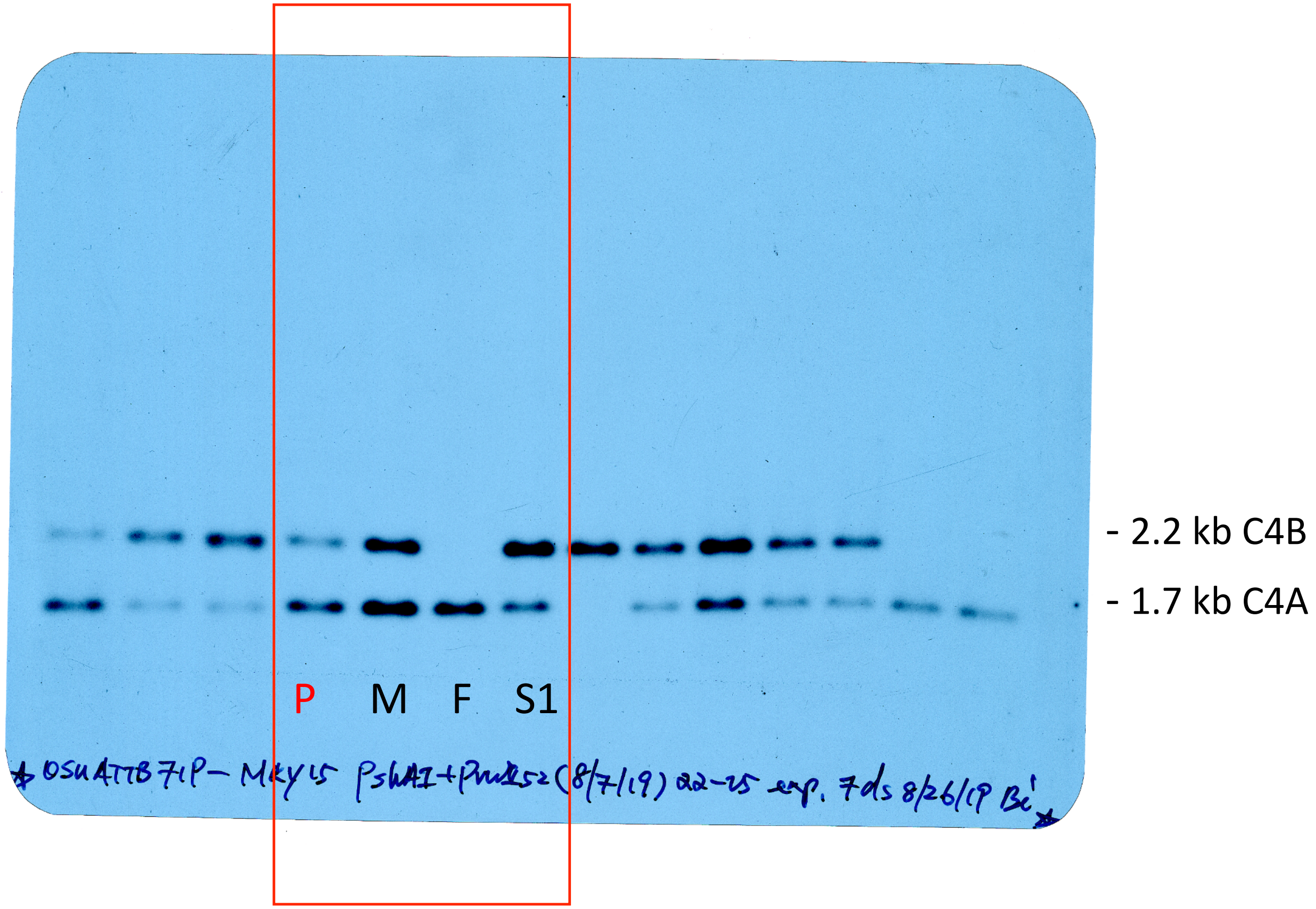

Original dried and stained immunofixed agarose gel

D. Immunofixation of EDTA plasma

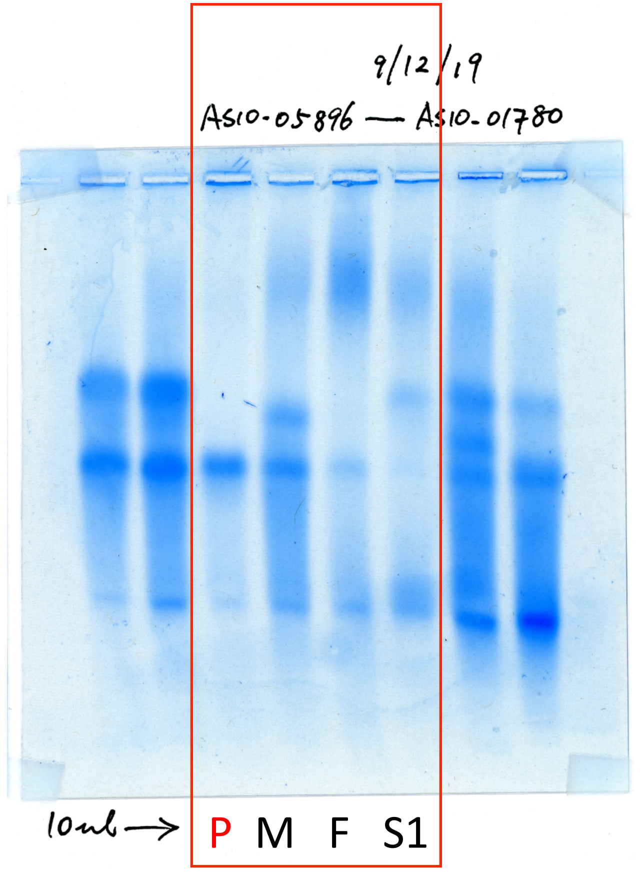

C4 protein aggregate

- C4B1 protein
- C4B2 protein
- C4A3 protein

Degraded C4 protein
